# Low-temperature open-air synthesis of PVP-coated NaYF_4_:Yb,Er,Mn upconversion nanoparticles with strong red emission

**DOI:** 10.1098/rsos.211508

**Published:** 2022-01-19

**Authors:** Lewis E. MacKenzie, Diana Alvarez-Ruiz, Robert Pal

**Affiliations:** ^1^ Department of Pure and Applied Chemistry, University of Strathclyde, Glasgow, UK; ^2^ Department of Chemistry, Durham University, Durham, UK; ^3^ GJ Russell Microscopy Facility, Department of Physics, Durham University, Durham, UK

**Keywords:** upconversion nanoparticles, polyvinylpyrrolidone, nanoparticle synthesis, near-infrared excitation, red emission, photonic upconversion

## Abstract

Cubic (α-phase) NaYF_4_:Yb,Er upconversion nanoparticles (UCNPs) are uniquely suited to biophotonics and biosensing applications due to their near-infrared excitation and visible red emission (*λ*_ex_ approx. 660 nm), enabling detection via thick overlying tissue with no bio-autofluorescence. However, UCNP synthesis typically requires high temperatures in combination with either high pressure reaction vessels or an inert atmosphere. Here, we report synthesis of α-phase NaYF_4_:Yb,Er,Mn UCNPs via the considerably more convenient PVP40-mediated route; a strategy that requires modest temperatures and relatively short reaction time (160°C, 2 h) in open air, with Mn^2+^ co-doping serving to greatly enhance red emission. The optimal Mn^2+^ co-doping level was found to be 35 mol %, which decreased the average maximum UCNP Feret diameter from 42 ± 11 to 36 ± 15 nm; reduced the crystal lattice parameter, *a*, from 5.52 to 5.45 Å; and greatly enhanced UCNP red/green emission ratio in EtOH by a factor of 5.6. The PVP40 coating enabled dispersal in water and organic solvents and can be exploited for further surface modification (e.g. silica shell formation). We anticipate that this straightforward UCNP synthesis method for producing strongly red-emitting UCNPs will be particularly beneficial for deep tissue biophotonics and biosensing applications.

## 1. Introduction

**Figure 1.**
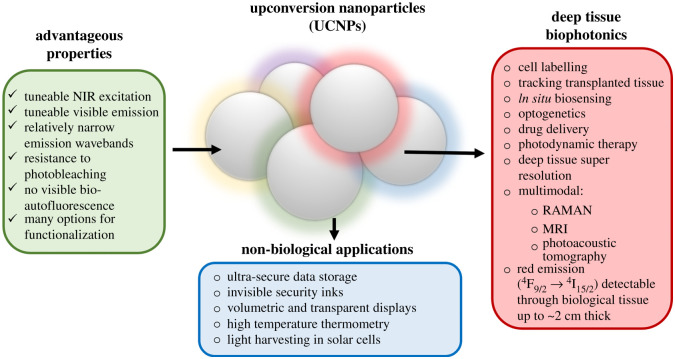
Overview of UCNP applications [4–20].

Upconversion nanoparticles (UCNPs) are uniquely suited for biophotonics and biosensing applications due to their unique low-energy near-infrared (NIR) excitation (*λ*_ex_ ∼ 980 nm) and visible emission (anti-Stokes' shift) at well-defined narrow wavebands. These properties enable deep tissue biological imaging without visible autofluorescence from biological components such as tissue and blood, and without the phototoxicity associated with conventional high energy UV/visible excitation [1]. These advantageous photophysical properties of UCNPs arise from their inorganic crystalline structure incorporating rare-earth dopants. For biomedical applications involving imaging through tissue, red-emitting cubic (α-phase) NaYF_4_:Yb,Er UCNPs are ideal because the excitation and emission of such UCNPs lies within the ‘near-infrared biological window’ (approx. 650–1000 nm), where optical absorption and scattering by biological tissues is minimal [2]. Further, UCNPs can exhibit good biocompatibility [3], and can be functionalized to enable many applications (figure 1). Notably, red emission from α-phase UCNPs can be imaged through approximately 2 cm of tissue when diffusely excited by NIR wavebands [21,22], allowing non-invasive detection of UCNP luminescence within deep tissues of small animal models [23].

**Figure 2.**
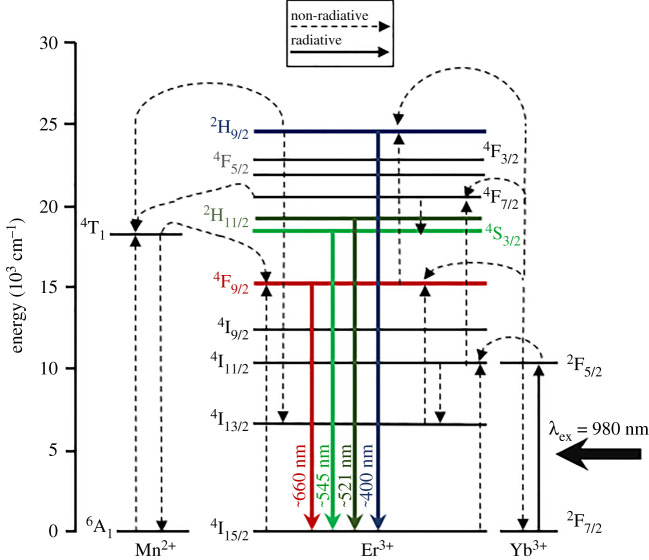
Energy level diagram depicting the proposed upconversion process. The 980 nm photons are first absorbed by multiple Yb^3+^ ions within the NaYF_4_ crystal lattice; energy is non-radiatively transferred to a single adjacent Er^3+^ ion via multi-ion Yb^3+^ to Yb^3+^ energy migration and then Yb^3+^ to Er^3+^ via an energy transfer upconversion process [26]. In Mn^2+^ co-doped UCNPs, red emission via the ^4^F_9/2_ level of Er^3+^ is enhanced by energy donation from the ^4^T_1_ level of neighbouring Mn^2+^ ions, which are in turned populated by non-radiative energy transfer from the high-energy ^4^F_7/2_ and ^2^H_9/2_ levels of Er^3+^. Back energy transfer and cross relaxation may also play a role but are not depicted here for simplicity [27].

The advantageous photophysical properties of UCNPs are determined by their rare earth ion dopant composition and crystalline lattice structure (see electronic supplementary material, figure S1). In NaYF_4_, Na^+^ and Y^3+^ cations provide a host lattice with ionic radii close to those of lanthanide dopants, while F^−^ ions contribute to the overall low photon energy of the NaYF_4_ lattice [24]. The asymmetrical crystal field of the NaYF_4_ host lattice also interacts with the partially filled 4*f* electron configuration of lanthanide dopants, enabling partial allowance of the otherwise Laporte-forbidden electron transitions, resulting in Förster dipole–dipole energy transfer between neighbouring lanthanide ions [25]. In a typical NaYF_4_:Yb,Er crystalline lattice, several low-energy NIR excitation photons at 980 nm are absorbed by multiple Yb^3+^ ions (the sensitizers) and transferred to a single emissive Er^3+^ ion (the emitter) via a non-radiative multi-ion upconversion energy transfer process (figure 2) [25,27]. For α-phase UCNPs, emission is biased towards the ^4^F_9/2_ → ^4^I_15/2_ transition of Er^3+^ (*λ*_em_ ∼ 660 ± 20 nm), whereas the more compact structure of hexagonal (β-phase) UCNPs favours emission via the ^2^H_11/2_ → ^4^I_15/2_ and ^4^S_3/2_ → ^4^I_15/2_ Er^3+^ transitions (*λ*_em_ ∼ 521 and 545 nm, respectively). Upconversion efficiency of α-phase UCNPs is less than β-phase UCNP upconversion (e.g. 2% versus 4%, respectively for powder samples) [24,28]. UCNP emission of all colours can be achieved by varying crystalline structure and incorporating various combinations of rare-earth dopants, such as Yb^3+^, Er^3+^, Ho^3+^, Gd^3+^, Pr^3+^, Sm^3+^, Nd^3+^, Ce^3+^, Dy^3+^, Tb^3+^ and Tm^3+^ [29]. Core–shell nanoparticle morphologies may also be exploited to modify photophysical properties, achieve wavelength-multiplexed excitation [30] and to mitigate solvent-induced quenching of the upconversion process [15,29,31–34].

UCNP synthesis presents some challenges which can limit rate and scale of production. The most commonly used UCNP synthesis procedures, i.e. hydro/solvothermal and thermal decomposition methods, require either an autoclave reaction chamber capable of withstanding elevated pressures (e.g. approx. 3 MPa) [35], or otherwise necessitate synthesis under inert oxygen-free atmosphere. Further, synthesis requires high-temperature reactions for extended durations, e.g. up to 24 h [35–39]. UCNP synthesis at approximately 200°C typically produces α-phase UCNPs, whereas synthesis at greater than approximately 300°C produces β-phase UCNPs (see electronic supplementary material, figure S1) [36]. These synthesis approaches are, therefore, time and labour intensive, making it particularly onerous to research the many possible UCNP co-doping permutations without automated assistance [29]. Further, β-phase UCNPs are particularly prone to forming larger nanorods [36,40], which are suboptimal for biological applications where small (less than 50 nm) spherical nanoparticles are optimal for cellular uptake [41–43]. In recent years, there have been efforts to develop very low-temperature synthesis procedures for upconversion nanophosphors; however, it is not clear if these particular nanophosphors retain their upconversion luminescence when dispersed in water or biological fluids [38,39]. Therefore, there is a clear need for a UCNP synthesis method which can produce UCNPs in a straightforward and convenient manner.

The polyvinylpyrrolidone (PVP)-mediated UCNP synthesis route—first reported by Li and Zhang in 2006—fulfils this need [44]. PVP is a biocompatible, relatively inert and stable polymer [45], which has both a strongly hydrophilic pyrrolidone moiety and a hydrophobic alkyl group (see electronic supplementary material, figure S2). The pyrrolidone moiety can coordinate with the positively charged Ln^3+^ and Na^+^ ions, enabling formation of UCNPs via controlled precipitation when F^−^ ions are introduced [46]. Ultimately, the polar nature of PVP also enables it to act as an amphiphilic surfactant, effectively constraining UCNP size by envelopment/encapsulation and enabling the PVP-coated UCNPs to be dispersed both in water and organic solvents [47]. Prior studies have shown that the PVP surface coating can facilitate the addition of a solid or mesoporous silica shell coating to protect the UCNPs from solvent quenching or to load other molecules [34,44,48]. To date, all studies using the PVP-mediated UCNP synthesis route have used PVP with an average molecular weight of 40 000 (PVP40). UCNPs are formed via reactions at 160°C for 2 h under normal atmosphere [5,44,48,49]]. Li & Zhang used this method to produce α-phase NaYF_4_:Yb,Er (20 mol% Yb, 2 mol% Er) UCNPs. Their UCNPs ranged in average diameter from 30 to 87 nm and exhibited approximately equal green and red emission wavebands (red/green emission ratio approx. 0.8) [44]. They also demonstrated that substitution of Er^3+^ to Tm^3+^ resulted in UCNPs with blue emission (*λ*_em_ ∼ 480 nm) [5,44]. The PVP40-mediated route for NaYF_4_:Yb,Er synthesis has been further developed for cellular and bacterial uptake applications by Sikora *et al*. [49] and Grüner *et al*. [48] with these studies showing somewhat greater red/green emission ratios (e.g. approx. 2 : 1 ratio for peaks at approx. 660 and 545 nm, respectively). Despite these successful demonstrations, the PVP40-mediated route has been largely overlooked for UCNP synthesis.

Several studies have demonstrated that reducing Y^3+^ content in favour of Mn^2+^ co-doping enhances red emission in a wide variety of UCNP structures and morphologies [22,40,50–54]. Perhaps the most striking examples of Mn^2+^ co-doping are provided by Tian *et al.* [40] and Zeng *et al*. [55] where hydrothermal UCNP synthesis reactions, which would otherwise form a mixture of nanoscale α-phase UCNPs and micrometre-sized β-phase NaYF_4_:Yb/Er UCNP rods, instead produces purely α-phase NaYF_4_:Yb,Er UCNPs with very strong red emission due to Mn^2+^ co-doping levels of approximately 30–40 mol% [40,55]. This dramatic change in UCNP morphology and photophysical properties has been attributed to the somewhat smaller size of Mn^2+^ ions compared with that of Y^3+^ ions (*r* = 0.81 versus 0.89 Å, respectively) favouring the cubic formation and inhibiting β-phase nanorod growth by introducing transient electric dipole effects [40]. Wang *et al*. [50] further demonstrated that Mn^2+^ co-doping enhanced the red emission of oleic-acid-capped NaYbF_4_:Er UCNPs synthesized via the hydrothermal method, with corresponding morphology changes from hexagonal UCNPs, to cubic UCNPs, to thin flake-like structures [50]. As a result of these impressive prior demonstrations, we hypothesized that incorporating Mn^2+^ into NaYF_4_:Yb,Er UCNPs synthesized via the convenient PVP40-mediated route would result in a convenient and straightforward method for producing UCNPs with strong red emission.

## 2. Experimental details

### 2.1. Reagents and materials

Yttrium(III) oxide [Y_2_O_3_] (99.99%); ytterbium(III) oxide [Yb_2_O_3_] (99.9%); erbium oxide [Er_2_O_3_] (99.9%); PVP 40 000 (PVP40) [(C_6_H_9_NO)x]; ammonium fluoride [NH_4_F] (greater than 99.99%); and sodium chloride (greater than 99.5% BioXtra) were purchased from Merck Life Science UK Ltd. Manganese(II) nirate tetrahydrate [Mn(NO_3_)_2_ · 4H_2_O] (98%); 70% nitric acid (HNO_3_) (laboratory reagent grade); ethylene glycol (EG) [(CH_2_OH)_2_] (greater than 99% extra pure ACROS Organics); and absolute EtOH (greater than or equal to 99.8%, analytical reagent grade) were purchased from Fisher Scientific. All materials were used without further purification.

### 2.2. Synthesis of PVP40-coated α-NaYF_4_:Yb,Er,Mn UCNPs

The PVP40-mediated UCNP synthesis method used herein was adapted from prior studies [44,48,49]. First, stock solutions of Y_2_O_3_, Yb_2_O_3_, Er_2_O_3_ were prepared in 10% nitric acid. These required preparation several days in advance due to the poor solubility of these lanthanide oxides at room temperature. Elevated temperatures were not used to assist dissolution in order to avoid thermal decomposition of nitric acid (clear colour) to nitrogen dioxide (yellow colour). Stocks of Mn(NO_3_)_2_ · 4H_2_O were prepared as required. Stock solutions of NH_4_F and PVP40 in ethylene glycol were prepared at room temperature at least 24 h in advance, with the PVP40 stock solution requiring vigorous manual stirring with a glass rod for 5 min.

UCNPs were prepared as follows. Mixture A: Y_2_O_3_, Yb_2_O_3_, Er_2_O_3_ and Mn(NO_3_)_2_·4H_2_O stocks in 10% HNO_3_ were combined in various amounts as per electronic supplementary material, table S1 to create a transparent solution containing a total of 1 mmol of Ln^3+^ and Mn^2+^ ions. This solution was then vigorously stirred for 1 min before heating at 120°C to evaporate the aqueous content, resulting in either a transparent residue (0 mol% Mn^2+^) or a brown residue (with Mn^2+^); the solution was removed from the heat when the residue stopped bubbling. A total of 8 ml of ethylene glycol was added, and the mixture was stirred at 80°C for a minimum of 30 min, or for as long as necessary for the solution to turn clear in colour. Once the solution was clear, 58.5 mg (1 mmol) of NaCl was added directly to the solution and stirred for 5 min at 80°C. Then 0.556 g/0.014 mmol of PVP40 stock (2 ml volume) was added dropwise and the mixture was stirred for 10 min at 80°C, after which the solution was transferred to a round bottom flask and maintained at 80°C via an oil bath. Mixture B was prepared by adding 8 ml of ethylene glycol to a conical flask and raising the temperature to 80°C. Then 4 mmol/148.2 mg of NH_4_F stock (2 ml volume) was added and the solution was stirred vigorously for 10 min. Mixture B was then added dropwise to Mixture A dropwise and vigorously stirred at 80°C for 10 min. The resultant solution was heated to 160°C via an oil bath at a rate of approximately 5°C min^−1^ and maintained at 160 ± 5°C for 2 h with vigorous stirring. As the reaction progressed, the solution clearly changed from semi-opaque and colourless to an opaque yellow/orange coloration, typically producing approximately 130 mg of PVP40-coated α-phase UCNPs. The UCNPs were collected by centrifugation at 10 000 relative centrifugal force (RCF) for 45 min. The supernatant was removed and replaced with 10 ml of EtOH. The UCNPs were then resuspended by sonication for 15 min. Two more wash steps were conducted (with centrifugation at 7000 RCF for 30 min), with final suspension of UCNPs in 5 ml of EtOH.

### 2.3. UCNP characterization

**Figure 3.**
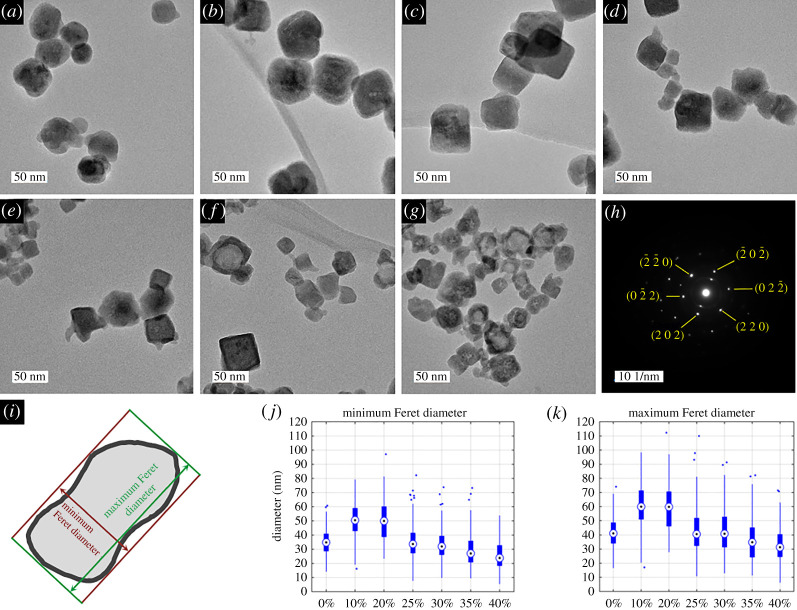
Mn^2+^ co-doping alters the size of UCNPs produced via the PVP40-mediated synthesis method. TEM images of synthesized PVP40-coated UCNPs (Yb^3+^ = 20 mol%, Er^3+^ = 2 mol%, Mn^2+^ = x mol%. Y^3+^ = 68 – x mol%) showing changes in UCNP morphology and diameter due to increasing concentration of Mn^2+^ co-doping. (*a*) 0 mol% Mn^2+^ UCNPs. (*b*) 10 mol% Mn^2+^ UCNPs. (*c*) 20 mol% Mn^2+^ UCNPs. (*d*) 25 mol% Mn^2+^ UCNPs. (*e*) 30 mol% Mn^2+^ UCNPs. (*f*) 35 mol% Mn^2+^ UCNPs. (*g*) 40 mol% Mn^2+^ UCNPs. (*h*) Selected area electron diffraction (SAED) pattern from the [111] zone axis of a single NaYF_4_:Yb,Er (20%,2%) (i.e. 0 mol% Mn^2+^) UCNP. (*i*) Example of minimum and maximum Feret diameter for an arbitrary nanoparticle. (*j*,*k*) boxplots of minimum and maximum Feret diameter distribution for each UCNP sample (*n* > 100 in all instances). Central marker = median diameter; top and bottom of box = 25th and 75th percentile ranges; whiskers = 0.7 and 99.3 percentile ranges; dots = outliers.

Transmission electron microscopy (TEM), electron dispersive spectroscopy (EDS) and selected area electron diffraction (SAED) were conducted as follows. Samples were prepared for imaging by sonicating the as-prepared UCNP solutions for 15 min and then adding 10 µl of UCNP sample to approximately 1 ml of isopropyl alcohol. This was then sonicated for 2 min, and a single drop was placed onto a holey carbon-supported copper grid and allowed to dry at room temperature for 24 h. TEM imaging was conducted on JEOL JEM-2100F operated at 200 kV with an Oxford Instruments 65 mm^2^ X-max X-ray detector for EDS measurements. UCNP diameter measurements were conducted manually with FIJI and tabulated in Microsoft Excel [56]. The minimum and maximum Feret diameter/calliper diameter (figure 3*i*) were analysed with a minimum of 100 UCNPs in each sample. For scanning electron microscopy (SEM), UCNP samples were prepared in a similar manner to TEM samples and 10 µl of each sample was gently dropped onto a pre-washed silicon wafer (Agar Scientific) and heated at 50°C using a hotplate to evaporate the isopropyl alcohol. Each silicon wafer was then mounted onto a stub mount and imaged with an SEM (Sigma 300 VP, Zeiss) using the InLens detector at 10 000×, 30 000× and 100 000× magnifications.

Dry powder UCNP samples (see electronic supplementary material, figure S3) were prepared for X-ray diffraction (XRD) and inductively coupled plasma mass spectroscopy (ICP-MS) measurements as follows. Approximately 10 mg of as-prepared UCNPs in EtOH was transferred to an open glass vial inside a fume hood and the EtOH was allowed to evaporate naturally. The resultant dry residue was then scraped within the vial to produce a loose white powder. For XRD measurements, the powdered sample was placed onto a silicon slide and held in place with a binding agent (Vaseline). Scans were conducted using a Bruker AXS D8 Advance GX 003 410 fitted with a Lynxeye Soller PSD detector and automated sample changer, and operated in Bragg–Brentano mode with a Ni filter. XRD patterns were acquired between 10 and 90° in 0.02° steps, with variable slit width fixed at 6 mm, and using a wavelength of 1.5406 Å (Cu *kα* source). The precise two-theta angle of each XRD peak was found by fitting a Gaussian profile to each peak using a custom-written analysis script (Matlab 2020b, Mathworks). The cubic (α-phase) crystal lattice parameter, *a*, was calculated from the average estimate from eight major via the following equations:

2.1 $$d_{hkl}=\frac{\lambda }{2\sin(2\theta /2)}$$

and

2.2 $$a_{hkl}\;=\;d_{hkl}\sqrt{h^{2}+k^{2}+l^{2}},$$

where *d_hkl_* is the interplanar spacing, *λ* is the X-ray wavelength, 2*θ* is the two-theta angle of each peak and *hkl* are the Miller indices corresponding to each peak.

Elemental composition was analysed by inductively coupled plasma atomic emission spectroscopy (ICP-AES) and inductively coupled plasma mass spectroscopy (ICP-MS). For these measurements a known mass of dry powdered sample was transferred to a perfluoroalkoxy alkane (PFA) vial. Then 3 ml of aqua regia was added, the vial sealed and heated to 100°C for 12 h. The sample was then diluted to 50 ml on the day of analysis. ICP-AES measurements were conducted using a Thermo Scientific SICAP 6000 previously optimized for sensitivity and signal stability. Multiple emission lines were measured for each element to monitor any spectral interference that may be present. ICP-MS measurements were conducted using a Thermo Scientific X-Series.

For luminescence measurements, 5.4 mg of as-prepared samples of UCNPs in EtOH were added to a 1 cm path length quartz cuvette (111-10-40 QS, Hellma) and the volume was adjusted to 0.9 ml by adding EtOH for a final UCNP concentration of 6 mg ml^−1^. The cuvette was placed inside an enclosed light-proof sample chamber for measurement. Excitation was provided by a femtosecond pulsed tuneable NIR laser (680–1300 nm, Coherent Discovery TPC, 100 fs, 80 MHz repetition rate) with a stated spot size of 1.2 mm and variable power output, with a typical maximum power of 1300 mW at 980 nm. The laser was routed to the sample chamber via beam routing mirrors (Thorlabs BB1-EO3) within light-tight tubes. The laser beam was focused onto the sample by an ultrafast 50 mm laser lens (11711, Edmund Optics) resulting in an approximate beam spot size of approximately 62 µm. Luminescence was measured 90° to excitation by an Ocean Optics HR2000Pro spectrometer (2048-pixel linear CCD Sony ILX5 chip, 200 µm slit, H3 grating, 350–850 nm spectral region). For standard luminescence intensity measurements, the excitation laser power was held at 70%, i.e. approximately 960 mW at 980 nm; 1000 spectra were acquired and averaged, with each spectrum being a 50 ms acquisition (i.e. approx. 50 s total acquisition time per measurement). UCNP samples were shaken gently before and after each measurement to ensure UCNPs were well suspended. The spectrometer was operated using a custom-written LabVIEW program (LabVIEW 2013). Emission was filtered by a less than 700 nm short-pass filter (84714, Edmund Optics) to ensure no stray excitation light reached the detector. Luminescence was analysed by custom Matlab scripts. Green emission intensity was quantified by the total emission area under the curve arising from the ^2^H_11/2_ → ^4^I_15/2_ (approx. 521 nm) and ^4^S_3/2_ → ^4^I_15/2_ (approx. 545 nm) emission transitions; red emission intensity was quantified as the total area under the emission curve of the ^4^F_9/2_ → ^4^I_15/2_ transition (approx. 660 nm). For measurements of UCNP luminescence versus excitation power, the UCNPs were suspended in ethylene glycol to avoid gradual sedimentation of UCNPs.

## 3. Results and discussion

**Figure 4.**
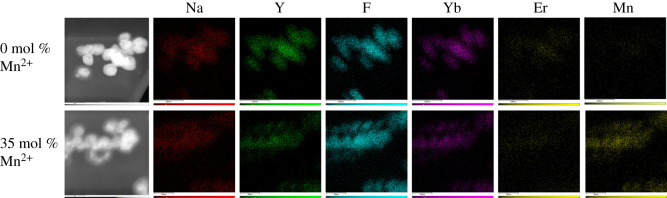
Elemental maps of 0 and 35 mol% Mn^2+^ UCNP samples obtained via energy-dispersive X-ray spectroscopy (EDS).

Representative TEM images of UCNPs produced are shown in figure 3*a–g*, with corresponding diameter distributions shown in figure 3*j,k*. UCNP diameter and aspect ratios are summarized in table 1. The NaYF_4_:Yb,Er (20 mol% Yb, 2 mol% Er) UCNPs produced (figure 3*a*) are very similar in form and diameter (maximum Feret diameter = 42 ± 11 nm) to UCNPs synthesized previously using the same method by Grüner *et al*. (diameter approx. 45 nm) [48], indicating that the PVP40 synthesis route constrains UCNP diameters in a reproducible manner. Addition of 10 mol% Mn^2+^ resulted in an increase of UCNP diameter to 60 ± 19 nm, but no change in overall UCNP aspect ratio. Similar results were obtained with 20 mol% Mn^2+^ co-doping. Notably, a Gaussian diameter distribution was observed for 0, 10 and 20 mol% Mn^2+^ samples. Increasing Mn^2+^ to 25 mol% produced UCNPs of a considerably smaller diameter (maximum Feret diameter = 43 ± 16 nm) and slightly lower aspect ratio. Notably, the diameter distribution of these UCNPs was lognormal instead of Gaussian. This trend continued for 30, 35 and 40 mol% Mn^2+^ (figure 3*e*,*f*,*g*,*j*,*k*), which produced smaller UCNPs (maximum Feret diameters = 43 ± 16, 36 ± 15 and 33 ± 13 nm, respectively). These smaller UCNPs were also less homogeneous in form (figure 3 and electronic supplementary material, figure S4) and slightly elongated (see aspect ratio results in table 1). SEM images (see electronic supplementary material, figure S5) confirm these UCNP morphology trends. Incorporation of Mn^2+^ within UCNPs was verified by qualitative EDS and quantitative ICP-AES/ICP-MS measurements (figure 4 and table 2).

**Table 1.** Summary of UCNP diameters.

mol% Mn^2+^	no. UCNPs analysed	diameter distribution	minimum Feret diameter (nm)			maximum Feret diameter (nm)			Aspect ratio min/max Feret diamete*r*
			mode (lognormal peak)	average	s.d.	mode (lognormal peak)	average	s.d.	
0	186	normal	—	35	9	—	42	11	0.835
10	132	normal	—	50	13	—	60	19	0.832
20	113	lognormal	44	50	14	52	59	17	0.834
25	229	lognormal	28	35	12	35	43	16	0.806
30	119	lognormal	27	33	12	34	43	16	0.774
35	218	lognormal	23	29	11	28	36	15	0.798
40	155	lognormal	20	26	10	26	33	13	0.769

**Table 2.** UCNP composition determined by ICP-AES (*) and ICP-MS (§). Uncertainties are calculated from the standard deviation of a minimum of three independent measurements from multiple emission lines or isotopes for each sample.

% weight	0 mol% Mn^2+^		35 mol% Mn^2+^	
	sample 1	sample 2	sample 1	sample 2
Na (*)	7.3 ± 0.3	7.4 ± 0.3	9.1 ± 0.3	8.7 ± 0.3
Y (*)	26.3 ± 2.4	31.3 ± 2.6	18.5 ± 1.0	17.8 ± 0.9
Yb (*)	14.4 ± 0.6	17.1 ± 0.7	17.4 ± 0.8	16.7 ± 0.8
Er (§)	2.2 ± 0.1	2.5 ± 0.1	2.7 ± 0.1	2.4 ± 0.1
Mn (*)	0.0 ± 0.0	0.0 ± 0.0	6.3 ± 0.2	6.1 ± 0.2

**Figure 5.**
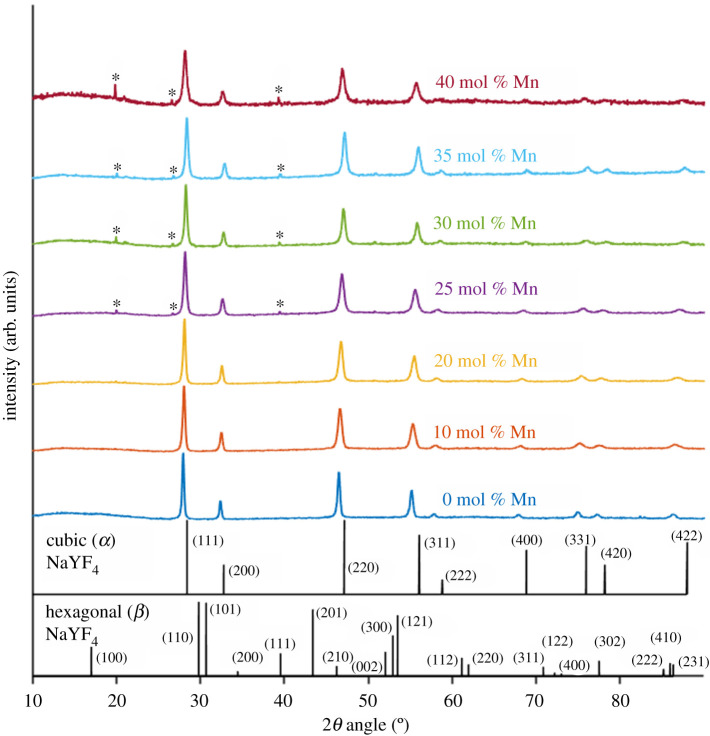
Powder XRD patterns obtained from each UCNP sample compared with reference patterns for cubic (*α*) and hexagonal (*β*) NaYF_4_. Peaks arising from increasing Mn^2+^ co-doping are denoted by *.

Powder XRD pattern analysis confirmed that UCNPs produced were cubic α-phase, with all lines produced by 0, 10 and 20 mol% Mn^2+^ UCNPs attributable to α-phase NaYF_4_ via comparison with test card data (figure 5). SAED from individual further confirmed that the 0 mol% Mn^2+^ UCNPs correspond to a well-structured crystal lattice (figure 3*h* and electronic supplementary material, figure S6). Acquisition of SAED measurements was attempted for 35 mol% Mn^2+^ UCNPs, but crystal planes suitable for analysis could not be identified. From powder XRD measurements, the α-phase crystal lattice parameter, *a*, was calculated to be *a* = 5.521 ± 0.003 Å for NaYF_4_:Yb,Er (20, 2 mol%) UCNPs. This value is identical to the value reported by Sikora *et al.* (5.52 Å) for UCNPs of the same composition synthesized with the same method [49], indicating consistent synthesis of UCNPs with this PVP40-mediated method.

Increasing Mn^2+^ mol% decreased the crystal lattice parameter, *a*, corresponding to a decrease in overall unit cell volume. The minimal value was *a* = 5.448 ± 0.002 Å for 35 mol% Mn^2+^ UCNPs (i.e. a 1.3% decrease from 0 mol% Mn^2+^ UCNPs). Intriguingly, *a* increased to *a* = 5.470 ± 0.007 Å for 40 mol% Mn^2+^ UCNPs. This trend was reflected in a shift towards higher 2*θ* angles for all samples between 0 and 35 mol% Mn^2+^, with a similar reversal for 40% Mn^2+^ (see electronic supplementary material, figure S8c,d,f,g,h). Similar shifts in 2*θ* angle have been reported for upconversion materials co-doped with Fe^3+^ and Mn^2+^ [55,57–60].

Three new minor XRD peaks were observed in UCNPs samples of Mn^2+^ co-doping greater than or equal to 25 mol% (figure 5 and electronic supplementary material, figure S8b and 8e). The peaks at 2*θ* = ∼20° and ∼39.5° may be attributable to (100) and (111) XRD peaks of β-phase NaYF_4_. However, the many other β-phase NaYF_4_ peaks did not arise. Further, a peak at 2*θ* = ∼26.7° arises which was not attributable to β-phase NaYF_4_ (see arrow in electronic supplementary material, figure S8c). Therefore, it is unclear as to exactly what crystal phase is formed at Mn^2+^ co-doping levels greater than or equal to 25 mol%. Other studies have postulated that incorporating Mn^2+^ into upconversion lattices otherwise occupied by Ln^3+^ can result in formation of F^−^ vacancies, which will alter the crystal lattice structure and induce lattice contraction, consistent with XRD observations [60]. With the data available to us, we cannot draw firm conclusions regarding the exact nature of the more complex unit cell structure formed with Mn^2+^ co-doping levels greater than or equal to 25 mol%. However, a candidate structure may be orthorhombic phase NaMn_3_F_10_ as previously inferred by Wang *et al*. [50].

**Figure 6.**
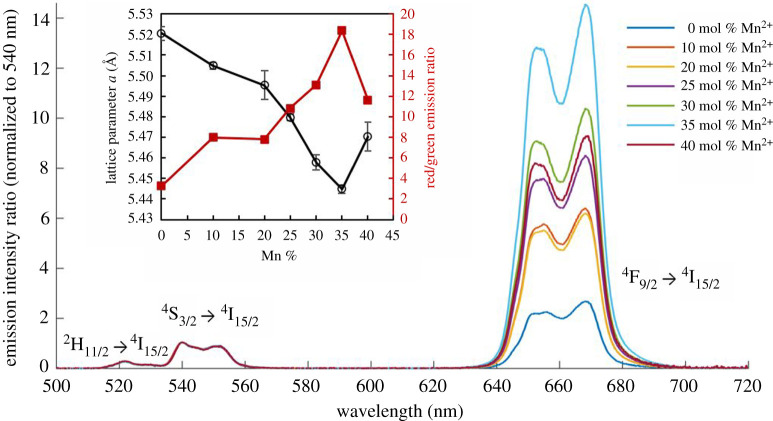
Red UCNP luminescence is enhanced due to the change in crystal lattice parameter induced by Mn^2+^ co-doping. Main figure: upconversion luminescence from UCNPs, 6.0 mg ml^−1^ in EtOH, *λ*_ex_ = 980 nm, normalized to intensity at 540 nm. Green emission arises from the ^2^H_11/2_ → ^4^I_15/2_ and ^4^S_3/2_ → ^4^I_15/2_ transitions. Red emission arises from the ^4^F_9/2_ → ^4^I_15/2_ transition. Inset: crystal lattice parameter, *a*, is strongly inversely correlated with UCNP red/green emission ratio (correlation coefficient = −0.97). Values calculated from eight XRD peaks; error bars = standard deviation.

Mn^2+^ co-doping enhanced red emission of the UCNPs (figure 6). For 0 mol% Mn^2+^ UCNPs, the emission spectrum was typical of α-phase UCNPs consisting of 20 mol% Yb^3+^ and 2 mol% Er^3+^, exhibiting a red/green emission ratio of 3.3. This emission spectrum is similar to those reported by prior studies of UCNPs synthesized via the PVP40-medited method [44,48,49], and corresponded to yellow emission on a CIE chromacity chart (see electronic supplementary material, figure S10). By contrast, a maximum red/green emission ratio of 18.4 was achieved for UCNPs co-doped with 35 mol% Mn^2+^, corresponding to red emission on a CIE chromaticity chart (see electronic supplementary material, figure S10). The red/green emission ratio of samples had a clear anti-correlation with the UCNP lattice parameter, *a* (correlation coefficient −0.97, figure 6 insert). This indicates that incorporating Mn^2+^ resulted in increased population of the ^4^F_9/2_ → ^4^I_15/2_ emission transition (*λ*_em_ = ∼660 nm). Notably, there was negligible emission from the ^2^H_9/2_ → ^4^I_15/2_ emission transition (*λ*_em_ = ∼410 nm), indicating that this higher energy transmission was not sensitized in any of the UCNPs produced.

**Figure 7.**
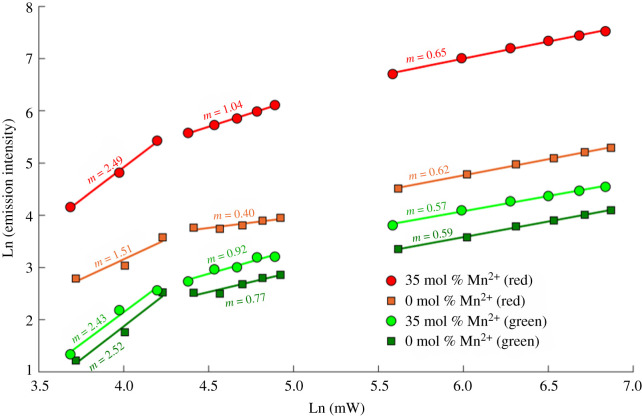
Excitation power dependence of upconversion luminescence for 0 mol% Mn^2+^ and 35 mol% Mn^2+^ UCNPs.

The multi-photon upconversion process was investigated by varying the excitation laser power and monitoring the emission of 0 mol% Mn^2+^ and 35 mol% Mn^2+^ UCNP in ethylene glycol (figure 7). In unsaturated upconversion processes, intensity of emission, *I*, scales as: *I* = *P^n^*, where *P* is the excitation power, and *n* is the number of photons required for upconversion. Therefore, the gradient of a log–log plot of excitation power versus UCNP emission corresponds to the number of photons involved in a non-saturated upconversion process. At high excitation powers, this process becomes saturated and the relation no longer holds, resulting in a gradient of less than 1 in such plots [61]. The most notable difference between samples is that the ^4^F_9/2_ → ^4^I_15/2_ emission pathway (*λ*_em_ = ∼660 nm) shows a two-photon dependence for 0 mol% Mn^2+^ UCNPs (m = 1.51), and a three-photon dependence for 35 mol% Mn^2+^ UCNPs (m = 2.49). This is consistent with the proposed upconversion process depicted in figure 2, where the ^4^F_9/2_ → ^4^I_15/2_ Er^3+^ transition is populated by energy transfer from ^4^T_1_ energy level of Mn^2+^ ions, which is in turn populated by the ^4^F_9/2_ energy level of Er^3+^ ions, and which is populated in turn by multi-photon upconversion from Yb^3+^ donors. This would explain why even modest Mn^2+^ co-doping resulted in increased red emission. Notably, emission via this transition was lessened for 40 mol% Mn^2+^ UCNPs, which may be indicative of non-radiative energy loss processes such as energy transfer between adjacent Mn^2+^ ions. The ^2^H_11/2_ → ^4^I_15/2_ and ^3^S_3/2_ → ^4^I_15/2_ emission pathways (*λ*_em_ = ∼521 and 545 nm respectively) demonstrate a three-photon power dependence for both 0 mol% Mn^2+^ and 35 mol% Mn^2+^ UCNPs. The UCNPs samples saturated at laser powers greater than 5% of maximum laser power, which due to the focused excitation beam, corresponded to an approximate power at the focal spot of 2150 W cm^−2^. This is similar to saturation processes observed for other upconversion materials [61–63].

Here we have shown the PVP40-mediated UCNP synthesis method can be used to create UCNPs with strong red emission via Mn^2+^ co-doping. Prior studies have shown that Mn^2+^ co-doping can dramatically improve red emission in a variety of UCNP morphologies produced via solvothermal and hydrothermal methods [22,40,50–55]. An important mechanism for this red emission improvement is that Mn^2+^ co-doping encourages formation of pure α-phase UCNP structure, which is well suited for red emission due to larger unit cell size than the smaller unit cells of β-phase UCNPs [40,55]. Further, intense single-band red emission has been achieved via Mn^2+^ co-doping resulting in suppression of the ^4^S_3/2_ → ^4^I_15/2_ (green) emission transition entirely in favour of the ^4^F_9/2_ → ^4^I_15/2_ (red) emission transition [40,64,65]. Whereas this study is the first to demonstrate that Mn^2+^ co-doping dramatically increases red emission in α-phase UCNPs produced via the relatively convenient PVP40 route without inducing an overall change in crystal phase. While pure single-band red emission was not achieved, multi-band red and green emission can be useful, e.g. for ratiometric sensing applications. The relative simplicity of the PVP40-mediated UCNP synthesis method makes it an attractive means of producing UCNPs without rate limitation requirements of hydrothermal/solvothermal pressure vessels. Yet the wider potential of the PVP40-mediated UCNP synthesis route has only barely been explored in publications to date [49,66,67]. Therefore further studies of the PVP40-mediated UCNP synthesis method and possible co-dopant combinations are warranted.

For example, completely forgoing the photodynamically inactive Y^3+^ ions within the host lattice in favour of Yb^3+^ sensitizer ions has been shown to considerably enhance the red emission of cubic NaYbF_4_:Er,Mn UCNPs synthesized via solvothermal methods [22]. Indeed, results from Sikora *et al*. indicate that increasing Yb^3+^ cation dopant concentration may improve population of the ^4^F_9/2_ → ^4^I_15/2_ emissive pathway for NaYF_4_:Yb,Er UCNPs synthesized via the PVP40 route [21,49]. Blue- and green-emitting PVP40-coated UCNPs can be synthesized by substation of Tm^3+^ instead of Er^3+^ as the emissive ion [5,44]. Fe^3+^ co-doping could also enhance red emission in α-phase UCNPs [55]. Other co-dopants such as Mo^3+^, Gd^3+^, Cu^2+^, Pr^3+^ and Ho^3+^ may also be of interest [29,59,68], with UCNPs incorporating Gd^3+^ being particularly notable for their use as dual-mode reporters for both optical luminescence and magnetic resonance imaging (MRI) [69]. While trialling UCNP dopant combinations is an inherently laborious process, the relatively straightforward and short PVP40-UCNP synthesis (approx. 2 h) lends itself well to trialling different UCNP compositions without resorting to automated aproaches [29].

The use of PVP for controlled synthesis of UCNPs could enable further tuning of UCNP properties by varying the molecular weight/chain length of the PVP used. For example, in seed-mediated Ag nanoparticle synthesis, the molecular weight/length of PVP chain used can dramatically alter the size and shape of nanoparticles produced [47]. It is also important to understand how PVP interacts with starting materials when forming UCNPs. For example, the lanthanide oxides used here have poor solubility in 10% nitric acid, so we postulated that it may be possible to form UCNPs from lanthanide nitrate hydrate starting materials (i.e. Y(NO_3_)_3_ · 6H_2_O, Yb(NO_3_)_3_ · 5H_2_O and Er(NO_3_)_3_ · 5H_2_O), which have excellent solubility in 10% nitric acid. Despite otherwise identical reaction conditions, these starting materials formed in inhomogeneous pseudo-nanorods with no apparent photonic upconversion activity (see electronic supplementary material, figure S13). ICP-MS results indicated that Na^+^ was not present in these samples, indicating a disrupted UCNP synthesis for unclear reasons, presumably due to the lanthanide nitrate starting materials. More promisingly, rare earth chlorates have been used as starting materials for PVP-mediated synthesis of green-emitting UCNPs for solar-cell applications [5], and so could provide further flexibility in choice of starting material. The long-term stability of PVP40-coated UCNPs in solution needs to be assessed because UCNPs coated with other polymers are known to gradually degrade in solution [70–72]. Prior studies have shown that PVP40 coating lends itself well to further UCNP modification via addition of solid or mesoporous silica shells, enabling protection from solvent quenching, drug loading and further polymer decoration for biocompatibility and bio-targeting [34,44,48].

## 4. Conclusion

We have demonstrated that PVP40-coated α-phase NaYF_4_:Yb,Er,Mn UCNPs with strong red emission can be produced in a convenient manner via the PVP40-mediated synthesis route. This method requires only simple hot plate and beaker apparatus and can be conducted under standard atmosphere at relatively low temperatures of 160°C with a relatively short 2 h primary reaction time. Mn^2+^ co-doping also altered unit cell composition with currently unidentified crystal lattice structures appearing at Mn^2+^ co-doping levels of greater than 25 mol%. Crucially, Mn^2+^ co-doping resulted in increased sensitization of the ^4^F_9/2_ → ^4^I_15/2_ Er optical emission pathway, with 35 mol% Mn^2+^ co-doping maximizing red emission, minimizing the unit cell size and producing overall smaller UCNPs with modified morphology and reduced aspect ratio. The UCNPs produced by this method are probably well suited to biomedical applications due to their strong red emission and average maximum Feret diameter of 36 ± 15 nm.

## Data accessibility

The supporting data for both this manuscript and associated supplementary material has been uploaded to the Dryad Data Repository under a Creative Commons Zero (CC0) licence and has been assigned the following doi:10.5061/dryad.qv9s4mwf.
